# Noncompaction of the Ventricular Myocardium Is Associated with a *De Novo* Mutation in the β-Myosin Heavy Chain Gene

**DOI:** 10.1371/journal.pone.0001362

**Published:** 2007-12-26

**Authors:** Birgit S. Budde, Priska Binner, Stephan Waldmüller, Wolfgang Höhne, Wulf Blankenfeldt, Sabine Hassfeld, Jürgen Brömsen, Anastassia Dermintzoglou, Marcus Wieczorek, Erik May, Elisabeth Kirst, Carmen Selignow, Kirsten Rackebrandt, Melanie Müller, Roger S. Goody, Hans-Peter Vosberg, Peter Nürnberg, Thomas Scheffold

**Affiliations:** 1 Cologne Center for Genomics and Institute for Genetics, University of Cologne, Cologne, Germany; 2 Institut für Herz-Kreislaufforschung, Universität Witten/Herdecke, Dortmund, Germany; 3 Institut für Biochemie, Charité-Universitätsmedizin Berlin, Berlin, Germany; 4 Abteilung Physikalische Biochemie, Max-Planck-Institut für Molekulare Physiologie, Dortmund, Germany; 5 Charité-Campus Berlin-Buch/Franz-Volhard-Klinik, Berlin, Germany; 6 Herzzentrum Duisburg, Duisburg, Germany; 7 Herzklinik am Augustinum, München, Germany; 8 Max-Planck-Institut für Herz- und Lungenforschung, Bad Nauheim, Germany; Stanford University, United States of America

## Abstract

Noncompaction of the ventricular myocardium (NVM) is the morphological hallmark of a rare familial or sporadic unclassified heart disease of heterogeneous origin. NVM results presumably from a congenital developmental error and has been traced back to single point mutations in various genes. The objective of this study was to determine the underlying genetic defect in a large German family suffering from NVM. Twenty four family members were clinically assessed using advanced imaging techniques. For molecular characterization, a genome-wide linkage analysis was undertaken and the disease locus was mapped to chromosome 14ptel-14q12. Subsequently, two genes of the disease interval, *MYH6* and *MYH7* (encoding the α- and β-myosin heavy chain, respectively) were sequenced, leading to the identification of a previously unknown *de novo* missense mutation, c.842G>C, in the gene *MYH7*. The mutation affects a highly conserved amino acid in the myosin subfragment-1 (R281T). *In silico* simulations suggest that the mutation R281T prevents the formation of a salt bridge between residues R281 and D325, thereby destabilizing the myosin head. The mutation was exclusively present in morphologically affected family members. A few members of the family displayed NVM in combination with other heart defects, such as dislocation of the tricuspid valve (Ebstein's anomaly, EA) and atrial septal defect (ASD). A high degree of clinical variability was observed, ranging from the absence of symptoms in childhood to cardiac death in the third decade of life. The data presented in this report provide first evidence that a mutation in a sarcomeric protein can cause noncompaction of the ventricular myocardium.

## Introduction

Cardiomyopathies are diseases of the heart associated with cardiac dysfunction of variable severity and prognosis. They have in the past been classified mainly based on morphological, but also on functional criteria. The terms “hypertrophy” or “dilatation” of the ventricles were used early to distinguish the most frequent types of cardiomyopathy. “Restriction” of ventricular filling and “arrhythmogenesis” with progressive loss of myocardial tissue described, in functional terms, the less frequent classes of cardiomyopathies. A generally accepted scheme of classifying these disorders was published by a World Health Organization (WHO) panel in 1995 [1]. Developments since then regarding newly discovered causes and advanced discrimination of phenotypes have caused an expert panel in the US to propose an update of previous definitions by stating that these diseases constitute “a heterogeneous group of myocardial disorders associated with mechanical and/or electrical dysfunction……due to a variety of causes that are frequently genetic” (for reference see [2]). These diseases have long been divided into “primary” and “secondary” cardiomyopathies. Primary cardiomyopathies (genetic or nongenetic) are predominantly confined to heart muscle. Secondary cardiomyopathies exhibit myocardial dysfunction in conjunction with a large variety of acquired or inherited systemic diseases. The case presented in this report belongs to the class of primary cardiomyopathies.

To briefly characterize the major members of this class, the most frequent disorder is “hypertrophic cardiomyopathy” (HCM [MIM 192600]) typically involving a hypertrophic, nondilated left ventricle often associated with left-ventricular outflow tract obstruction. Heart failure symptoms-with a relatively high risk of sudden cardiac death-develop usually in young adults or later in life. HCM has been traced to a variety of mutations in genes encoding contractile proteins of the cardiac sarcomere. Most commonly affected are the genes for ß-myosin heavy chain and for myosin-binding protein C. Nine other sarcomeric genes contribute less frequently to HCM. More than 250 different autosomal dominant mutations (probably many more, not all are published) have been identified in patients and families (for a recent review see [3]).

A typical feature of “dilated cardiomyopathy” (DCM [MIM 115200]) (with genetic or nongenetic causes) is ventricular chamber enlargement linked to systolic dysfunction. Clinical hallmarks are progressive heart failure due to a gradual loss of left-ventricular contractile function coupled with a high risk of sudden or heart failure-related death. Up to 35% of DCM cases have been reported as familial linked to more than 20 genomic loci and genes. The predominant mode of transmission is autosomal dominant. X-linked, autosomal recessive or mitochondrial inheritance are less frequent. Several mutated genes code for the same contractile proteins that otherwise cause HCM. Other genes encode Z-disc binding proteins, sarcolemmal and nuclear envelope components and transcriptional coactivators. Regarding genetic causes, overlap exists not only with HCM, but also with X-linked cardioskeletal myopathies in children (for a recent review see [4]).

“Arrhythmogenic right-ventricular cardiomyopathy/dysplasia” (ARVC/D [MIM 609040], a rare disorder) has a broad clinical spectrum usually presenting with ventricular tachyarrhythmia, syncopes, even cardiac arrest. This condition is often not easily diagnosed. A sensitive marker is a biopsy from the right ventricle exposing fibrofatty infiltrations with interspersed strands of myocytes. Inheritance is autosomal dominant in most cases. Four genes associated with ARVC/D have been detected involving intermediate filaments and desmosomes and the calcium release channel RyR2 in the myocardial sarcoplasmic reticulum (for a recent review see [5]).

“Noncompaction of (left-) ventricular myocardium” (NVM, also designated LVNC [MIM 300183]), a recently recognized congenital cardiomyopathy, shows typically a “spongy” appearance of the left-ventricular myocardium. NVM was first described in 1984 [6]. Albeit rare, it is now due to improved diagnostic technology recognized more frequently than previously. Both, syndromic and isolated cases have been identified [7]–[9]. Clinical consequences are variable. Early onset of symptoms has been reported [9], but others described long latency with presentation in adults at an advanced age, generally including congestive heart failure, ventricular arrhythmias and systemic thromboembolism [7], [10]–[12]. The myocardium of affected individuals is characterized by persisting trabeculations and deep intertrabecular recesses, reminiscent of the cardiac muscle in the developing heart. Accordingly, noncompaction of the ventricular myocardium has been suggested to result from an intrauterine arrest of cardiac development [7], [13].

In family studies evidence has been obtained for genetic heterogeneity of NVM. X-chromosomal causes have been linked to the *TAZ* gene encoding taffazin [MIM 300394], also associated with the Barth syndrome in children [MIM 302060] [9]. Autosomal mutations have been identified in *LDB3*, the LIM domain-binding protein 3 gene on chromosome 10 [MIM 605906] and in *DTNA* on chromosome 18 [MIM 601239] [10]. *LDB3* encodes ZASP, the ‘Z-band alternatively spliced PDZ motif-containing protein’ [14]. *DTNA* encodes α-dystrobrevin. Other investigations pointed to additional NVM loci with unknown genes on chromosome 5 and 11, respectively [15], [16].

Here we report on the results of a clinical and genetic analysis of a large German family suffering from NVM. The detailed clinical and morphologic characterization of the members of family DU-11 afforded the identification of a disease-related genomic locus on chromosome 14q by a genome-wide linkage analysis. Further refinement of the disease gene region led to sequencing of two genes, *MYH6* [MIM 160710] and *MYH7* [MIM 160760], encoding the α- and β-myosin heavy chain, respectively. A previously unknown missense mutation was detected in the gene encoding the β-myosin heavy chain (*MYH7*), the predominant myosin isoform in the ventricular myocardium. Finally, via an *in silico* analysis clues to structural and functional consequences of the mutation in the β-myosin heavy chain were obtained.

## Results

### Clinical Findings

**Figure 1:**
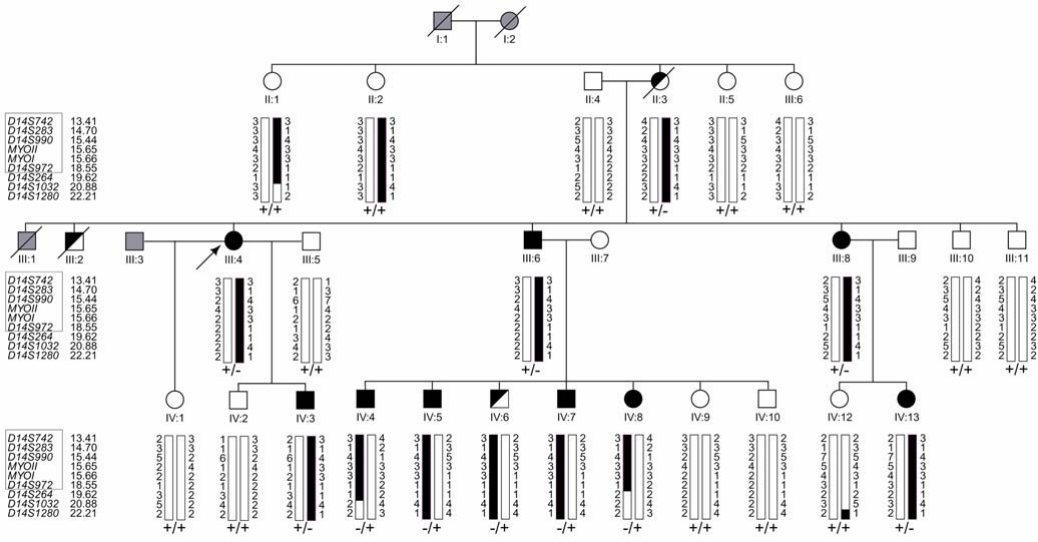
Pedigree of family DU-11 with haplotypes on chromosome 14 and segregation of *MYH7* mutation R281T (c.842G>C). Family members are shown by filled black symbols (affected), by half filled symbols (partially affected), open symbols (unaffected) and gray symbols (unknown affection status). An arrow points to the index patient. The disease associated haplotype is shown by filled black bars. Markers within the minimum disease associated haplotype are boxed. Below the haplotypes of each individual the occurrence of wild-type (+) and mutant *MYH7* alleles (−) is indicated.

**Figure 2:**
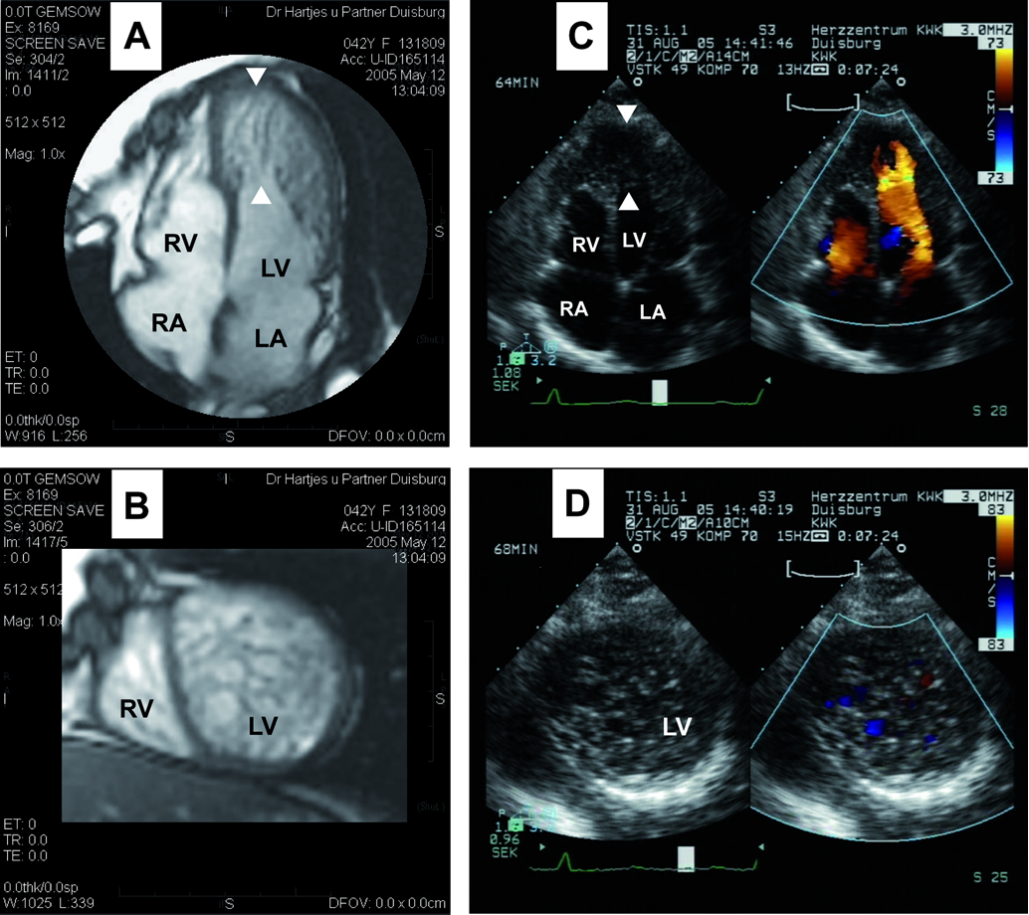
Imaging of NVM in patient III:8 of family DU-11. A, Magnetic resonance image: long-axis plane in diastole. Prominent myocardial trabeculations and deep intertrabecular recesses are seen in the apical half of the right and left ventricle as indicated by arrows. B, Magnetic resonance image: short-axis plane in diastole. In the apex region the cavum and the myocardial wall can not clearly be distinguished. Extensive trabeculations in this region produce a sponge-like appearance of the myocardium. C, Two-dimensional echocardiograph: apical four-chamber view in diastole with (right) and without (left) color flow imaging. A massive apical thickening is seen without any evidence of trabeculations in the left image. In contrast, color flow imaging (right image) shows more clearly the recesses extending deeply into the myocardial wall. D, Two-dimensional echocardiograph: parasternal short-axis view in diastole with (right) and without (left) color flow imaging. The left image shows a mesh-like morphology in the mid-region of the left ventricle. The cavum is not clearly discernable. The right image demonstrates numerous small blood-flow eddies within the sponge-like myocardium (as shown by color flow imaging). LV, left ventricle; RV, right ventricle; LA, left atrium; RA, right atrium.

**Figure 3:**
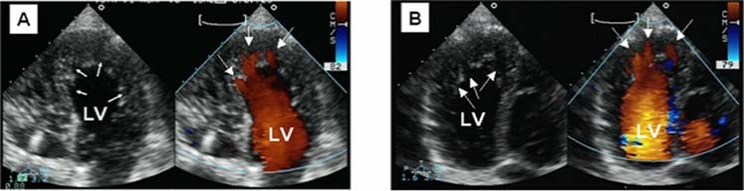
Imaging of NVM in the index patient (III:4, panel A) and her sister's daughter (IV:13, panel B). Shown are two-dimensional echocardiographs (ventricular part of a four-chamber view) in diastole, with (right) and without (left) color flow imaging. Arrows point to recesses in the myocardial wall.

In order to identify all cases of noncompaction within the family DU-11, transthoracic echocardiography (TTE) was employed (2D mode, M mode and color Doppler imaging). NVM was observed in three generations (fig. 1). The morphological phenotype observed varied from apical thickening with mild trabecularization to a spongy myocardium affecting the apical half of the left ventricle. The sponge-like appearance of the apical myocardium with excessive trabeculations in apex regions of both ventricles is shown in figure 2. NVM was further confirmed by TTE in a total of 9 patients (see table 1 and fig. 3 for echocardiographs of the index patient and her sister's daughter). Two deceased individuals (II:3 and III:2) and one living child (IV:6) were considered partially affected (see Materials and Methods).

**Table 1:** Patient Characteristics (initial presentation)

Patient	Age^a^	Sex	Clinical Status^b^	EA^c^	ASD^d^	aECG^e^	NVM^f^
II-3	dc	F	HF-NYHA III	+	+	+	(+) non-isolated
			dc at 61 years				
			due to sepsis				
III-2	dc	M	CD at 26 years	+	+	+	(+) non-isolated
III-4	39	F	HF-NYHA III-IV	+	+	+	+ non-isolated
III-6	38	M	asymptomatic	-	-	-	+ isolated
III-8	36	F	HF-NYHA I	-	-	-	+ isolated
IV-3	13	M	asymptomatic	+	-	+	+ non-isolated
IV-4	18	M	asymptomatic	-	-	+	+ isolated
IV-5	14	M	asymptomatic	-	-	+	+ isolated
IV-6	11	M	asymptomatic	-	-	+	(+) isolated
IV-7	10	M	asymptomatic	-	-	+	+ isolated
IV-8	7	F	asymptomatic	-	-	+	+ isolated
IV-13	7	F	asymptomatic	-	+	+	+ non-isolated

a Age at presentation in years; dc, deceased;

b HF, chronic heart failure; NYHA, classi-fication of heart failure according to the New York Heart Association; CD, cardiac death

c EA, Ebstein's anomaly;

d ASD, atrial septal defect;

e aECG, abnormal ECG;

f NVM, non-compaction of the ventricular myocardium; +, affected; (+), partially affected; see Material and Methods for details on the affection status; non-isolated/isolated: with/without EA and/or ASD. Listed are all carriers of the *MYH7* mutation and in addition individual III-2 who was not genotyped.

In addition to NVM, three individuals displayed an atrial septal defect (ASD [MIM 108800]) and Ebstein's anomaly (EA, [MIM 224700]). One patient had NVM and ASD and one patient showed NVM in combination with EA. All probands showed normal thickness of the interventricular septum and of the free ventricular wall. Signs of a canonical hypertrophic cardiomyopathy, such as asymmetric hypertrophy or thickening of the interventricular septum, were not observed.

NVM in family DU-11 was generally associated with a hypokinetic apex (as opposed to the hyperkinetic apex associated with apical HCM). As a consequence, the pump function was reduced (see below). In contrast to the typical picture of dilated cardiomyopathy, the end-diastolic diameter was below 55 mm in all adult probands.

The index patient (III:4) presented at the age of 39 years with dyspnoea upon exertion. Her condition and the condition of her sister (III:8) deteriorated within 3 years after initial presentation, resulting in a severely reduced pump function (2.4 and 2.1 l/min, respectively , measured using thermodilution; normal range: 4.0–10.2 l/min). They are currently enlisted for heart transplantation. Cardiac symptoms were also reported from the index patient's mother (II:3) and from her sister's daughter (IV:13). Patient II:3 suffered from an atrio-ventricular block (AV-block) which required implantation of a pacemaker. She died from sepsis related to a pacemaker-associated infection. The clinical status of individual IV:13 changed from asymptomatic to NYHA I (classification of heart failure according to the New York Heart Association) during follow-up. Two cases of premature death were reported from the third generation. One member of the family died as an infant from unknown cause (III:1), and another one as a young man at the age of 26 years (III:2). Patient III:2 suffered from a hemodynamically relevant defect of the atrial septum, recurrent syncopes and cardiac death. Eight family members were clinically asymptomatic at presentation, but had clear morphological signs of noncompaction. Regarding clinical severity, there is a tendency of NVM to develop unfavorably in adult family members. Based on the variable and age-dependent clinical expressivity we considered a reduced penetrance of the phenotype for further genetic analysis.

### Linkage of NVM to chromosome 14q

**Figure 4:**
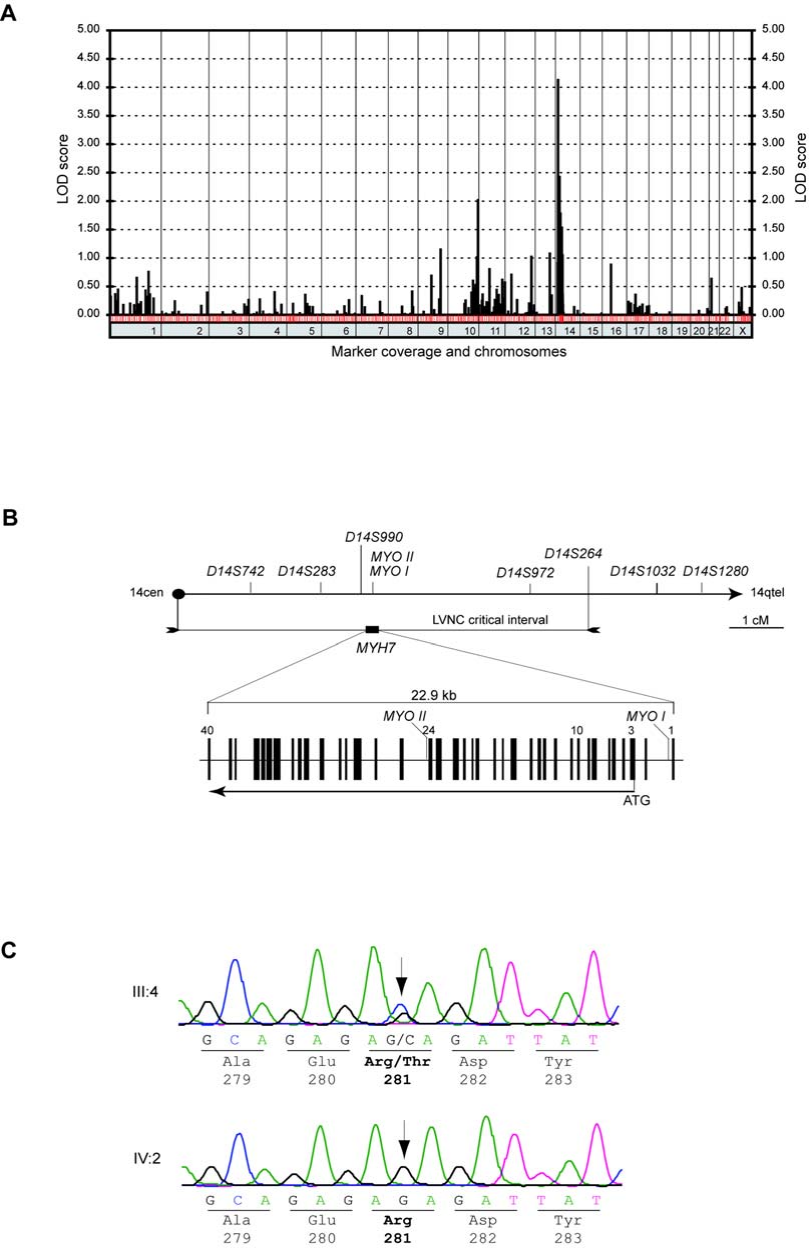
Genetic analysis. A, Linkage analysis. Two-point LOD scores for all chromosomes under the assumption of a reduced penetrance of 90% are shown. In the line below the diagram each red slash indicates the position of an analyzed marker on the corresponding chromosome. B, Genetic map of the candidate region 14cen-14q12. A part of the deCODE map is shown in the upper part. The critical interval is delimited by 14ptel and *D14S264*. The genomic organization of *MYH7* is delineated in the lower part. Exons are indicated by filled boxes and introns by horizontal lines. The translational start codon is located in exon 3. The positions of the intragenic markers *MYO* I in intron 1and *MYOII* in intron 24 are shown. The mutation was identified in exon 10. C, Mutation analysis. Chromatograms of the index patient III:4 and her unaffected son IV:2 are shown. The mutation site is marked by an arrow. Parts of the nucleotide sequence (from c.835 to c.849) and protein sequence (from 279 to 283) are given below.

In previous family studies, evidence has been obtained for genetic heterogeneity of NVM[9], [10], [14]–[16]. Two of the proteins involved, ZASP and α-dystrobrevin, are linked to the myocardial cytoskeleton. To identify the locus underlying NVM in family DU-11, we performed a genome-wide linkage analysis using microsatellite markers at a spacing of about 10 cM. We used morphological changes as determined by echocardiography to define a total of 9 affected, 2 partially affected and 13 unaffected family members, which we all included into the genome scan, and found significant linkage to a locus on chromosome 14. Under the assumption of a dominant mode of inheritance with a reduced penetrance of 90% for the affected individuals and of 50% for the partially affected individuals (II:3 and IV:6) a maximum two point LOD score of 4.15 was obtained for marker *D14S990* at φ = 0 (fig. 4A). Calculations under the same assumptions but with a penetrance of either 80% or 70% resulted in slightly reduced maximum LOD scores of 3.82 and 3.53 for marker *D14S990* at φ = 0, respectively (data not shown). We saturated the critical region on chromosome 14 with additional markers and constructed haplotypes. Obligate recombinants revealed a minimum common disease haplotype, i.e. shared by all patients, ranging from 14ptel to marker *D14S264* (fig. 1 and fig. 4B). The finding that proband II:3 was clinically affected, while the two sisters who shared the same haplotype (probands II:1 and II:2) were not, allowed to tentatively conclude that individual II:3 was a carrier of a *de novo* mutation. In this context it is worth to note that neither of the two unaffected sisters had children which could have been analyzed for the presence or absence of NVM. A LOD score of 3.82 (or 3.53) for the marker *D14S990* led us to suppose a mutation in either of the two myosin heavy chain genes *MYH6* or *MYH7*, both mapping to this region of chromosome 14. To test this suggestion, the two genes coding for α- and β-myosin heavy chain were subsequently sequenced.

### A missense mutation in *MYH7* cosegregates with NVM

The defined critical region includes the genes *MYH6* and *MYH7* encoding the cardiac α- and β-myosin heavy chains (MHC-α and MHC-β), respectively. Since MHC-β is the predominant isoform in the human ventricle [17], [18], we decided to investigate in detail *MYH7* as a likely candidate gene. By sequencing the 40 exons of *MYH7* in the index patient a heterozygous single nucleotide exchange at the position c.842G>C in exon 10 was identified. As a consequence, a positively charged arginine is replaced by an uncharged threonine residue at position 281 of MHC-β (R281T) (fig. 4C). As found by pyrosequencing and restriction fragment length polymorphism analysis, this substitution is present in all family members affected by NVM but absent from all unaffected family members. Thus, a perfect cosegregation between the NVM phenotype (affected and partially affected) and the R281T mutation was observed in our family. The absence of both the mutation and noncompaction in two siblings who carry the same “disease” haplotype as their affected sister (II:3) may be explained by a germ line mutation in one of the parents. The occurrence of a *de novo* mutation in patient II:3 would provide a further strong argument in favor of the disease-causing nature of this mutation. To exclude a neutral polymorphic variant, 184 healthy control individuals were tested negative for the exchange at position c.842 of *MYH7*. Further, it is unlikely that two other disease-causing genes, *LDB3* (encoding Cypher/ZASP) and *DTNA* (encoding α-dystrobrevin), contribute to noncompaction observed in family DU-11 since no linkage was found with either of these loci. Finally, sequencing of the gene *MYH6* did not reveal any missense or truncation mutation in the α-myosin heavy chain gene (data not shown). In a nutshell, we have detected a new, hitherto unknown mutation in exon 10 of *MYH7*, responsible for a phenotype previously not linked to this gene.

### Assumed consequences of the R281T mutation

**Figure 5:**
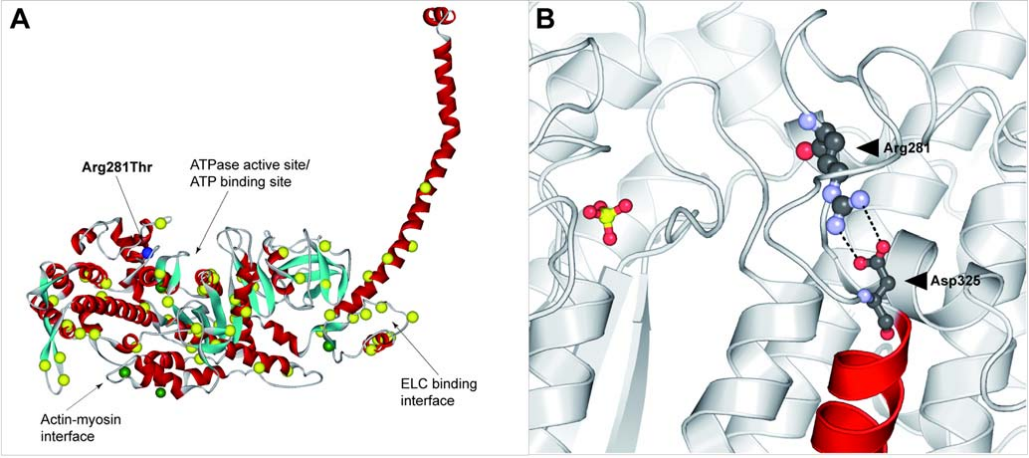
Localization of R281 in a protein model of the myosin heavy chain. A, Positions of amino acids affected in NVM, HCM and DCM. The mutations were plotted on a model of chicken skeletal myosin subfragment-1 (PDB code 2MYS). Functional sites are indicated by arrows. 68 selected mutations causing HCM are indicated by yellow spheres and 4 mutations causing DCM by green spheres. The mutations were selected from the UniProt database (UniProtKB) and from two publications[33], [34]. The described NVM causing mutation is highlighted as a blue sphere and labeled according to the position in MYH7_HUMAN. B, Close-up view of the salt bridge between residues R281 and D325 in chicken skeletal myosin subfragment-1 (PDB code 2MYS). The amino acids are numbered according to MYH7_HUMAN. The sulfate molecule (yellow and orange) marks the ATPase active site for better orientation. The residues R281 and D325 are shown according to the CPK color scheme (grey, carbon atoms; red, oxygen atoms, i.e. acidic side chain; light blue, nitrogen atoms, i.e. basic side chain). The salt bridge between R281 and D325 is symbolized by dashed black lines that indicate potential hydrogen bonds. The helix attached to D325 is highlighted in red.

**Figure 6:**
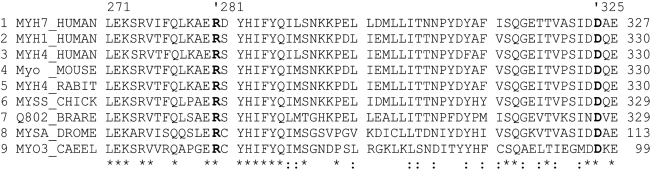
Multiple alignment of different myosin molecules. The interacting sites R281 and D325 (both in bold, position numbering according to MYH7_HUMAN) are highly conserved between myosin molecules of human, fruit fly, zebrafish and *Caenorhabditis elegans*, respectively (*, identical position; :, conservative exchange). Sequence names correspond to SwissProt entry names: MYH7_HUMAN-human myosin heavy chain, cardiac muscle beta isoform; MYH1_HUMAN-human myosin heavy chain, skeletal muscle, adult 1; MYH4_HUMAN-human myosin heavy chain, skeletal muscle, fetal; MYH1_MOUSE-murine myosin heavy chain, skeletal muscle, adult 1; MYH4_RABIT-rabbit myosin heavy chain, skeletal muscle, juvenile; MYSS_CHICK-chicken myosin heavy chain, skeletal muscle, adult; Q802Z4_BRARE (Q802_BRARE)-zebrafish protein Q802Z4; MYSA_DROME-fruit fly myosin heavy chain, muscle; MYO3_CAEEL-*Caenorhabditis elegans* myosin heavy chain A.

β-myosin is one of the principal constituents of the contractile apparatus of ventricular myocytes. It consists of two heavy chains and two pairs of light chains. The subfragment-1 (S1) contains the ATPase site and an actin binding region (fig. 5A), both are crucial for the movement of actin relative to myosin[19]. The R281T missense mutation is located in the globular head of MHC-β and affects a position in the vicinity of the switch I loop of the ATPase site[19]. R281 is highly conserved in myosins, with a large evolutionary distance ranging from insects (*D. melanogaster*) over invertebrates to humans (fig. 6). It is therefore possible to discuss the consequences of the R281T exchange by referring to crystal structures of orthologues of human MHC-β, e.g. the S1 domain of chicken gizzard myosin (PDB entry 2MYS, approximately 75% sequence identity with S1 of human MHC-β)[19]. In this structure, the position corresponding to human R281 (chicken R283) is involved in a salt bridge with a highly conserved aspartic acid residue (human D325/chicken D327) (fig. 5B). The salt bridge is buried below the protein surface and anchors an α-helix within a saddle-shaped structural protrusion on the surface of S1 (residues 275 to 330, approx.). Replacing either R281 or D325 will lead to the destabilization of this subregion within S1. Hence it is likely that a mutation of arginine 281 to threonine affects the normal folding and/or the ATPase activity of β-myosin, leading to altered contractile function of the sarcomere.

We do not know at present why a mutation in a contractile protein can culminate in a similar condition like mutations that affect cytoskeletal components. Further, it remains unclear, why R281T–in contrast to all other known *MYH7* mutations–causes NVM rather that HCM or DCM. Mutations associated with HCM and DCM are clustered in four functionally important regions: the actin-myosin interface, the ATPase active site with the ATP binding region, the essential light chain (ELC) binding interface and the rod (fig. 5A) [20]. In contrast to all other HCM or DCM mutations known so far, the R281T substitution is localized in exon 10. Hence, it will be interesting to see whether other mutations in the same exon cause a similar phenotype.

## Discussion

In the present study, we have linked noncompaction of the ventricular myocardium to a mutation in the β-myosin heavy chain gene. Despite the fact that a typical “HCM/DCM gene” was affected, none of the mutation carriers showed signs of canonical hypertrophic or dilated cardiomyopathy. Rather, some–but not all–mutation carriers had congenital heart disease, namely Ebstein's anomaly, i.e. a dislocation of the tricuspid valve, and/or an atrial septal defect. Thus, the R281T substitution was linked to either an isolated or non-isolated form of NVM that–in this family–did not develop into HCM or DCM.

The clinical consequences of the R281T mutation were diverse. Symptoms were generally absent in childhood and adolescence. One case of cardiac death in early childhood (III:1) may have been related to disease transmitted in this family, but no data exist. In two sisters of the third generation (III:4 and III:8) symptoms deterioated rapidly in the third and fourth decade of life, while one of their brothers (III:6) was at initial presentation asymptomatic, but he was identified as affected by noncompaction and also as carrier of the *MYH7* mutation. (Note, this proband later declined follow-up investigations.) An older brother of the two affected sisters (III:2) died (in the 80s) from cardiac death at age 26. No clinical data as to details of his cardiac conditon are available, nor was he genotyped. It does not seem unlikely to us, however, that he was also a carrier of the mutation detected in his first-degree sibs. Noncompaction was in our family clearly identified in early childhood. The youngest NVM carrier was 7 years old at the time of diagnosis. Hence, in agreement with others we conclude that NVM was present at birth [12]. Otherwise, symptoms developed later in life, strongly suggesting an age-dependent penetrance of the condition in family DU-11.

We hypothesize, that two nosological effects may be superimposed in the affected members of the family. One of them, noncompaction may have mild consequences, at least early in life. The second effect may result from the suggested structural change of the cardiac myosin heavy chain which possibly leads to an altered contractile function causing phenotypic consequences later in life. If a myosin-related dysfunction was involved, it did, however, not induce myocardial hypertrophy or chamber dilatation, the structural hallmarks of HCM and DCM, respectively. Congenital malformations such as Ebstein's anomaly or atrial septal defect, as seen in some family members, may have further contributed to the symptoms.

A key question is whether a mutation in a contractile protein (such as the β-myosin heavy chain) can trigger defects in both compaction of the myocardium and septation of the atria. In this context it is noteworthy that our results are essentially in line with three recent reports demonstrating, that mutations in myosin heavy chain genes can have damaging effects on the cardiovascular development in humans. A missense mutation, I820N, in *MYH6*, which codes for predominantly atrial α-myosin heavy chain (MHC-α) results in incomplete atrial septum formation. A similar effect was observed in developing chicken atria upon knock-down repression of *MYH6* using oligonucleotides complementary to *MYH6* mRNA, suggesting that an altered MHC-α structure or lack of this protein contribute to congenital heart disease [21]. Since the β-isoform of myosin heavy chain is also present in atria, albeit at rather low levels [22], one has to consider the possibility, that a mutation in *MYH7* can also provide the basis for the development of ASD. Secondly, in a separate study, various mutations in *MYH3* [MIM 160720] encoding embryonic myosin heavy chain have been causally linked to the occurence of the Freeman-Sheldon syndrome [MIM 193700] and the Sheldon-Hall syndrome (alternatively designated type 2B distal arthrogryposis [MIM 601680]). These syndromes are characterized by congenital manifestation of severe isolated or multiple contractures. It was proposed, that dysfunctional force production in myofibers in an early stage of embryogenesis was responsible for these symdromes [23]. In a third study, mutations in *MYH11* [MIM 160745], the gene coding for smooth muscle (SM) myosin heavy chain were shown to trigger patent ductus arteriosus as a congenital trait and the formation of thoracic aortic aneurysms or aortic dissections in adults. A dominant negative effect was strongly suggested to be due to improper coiled-coil formation of the SM myosin rod [24]. Taken together, these data argue in favor of a general role of the myosin heavy chain in the development of the cardiovascular system.

In conclusion, our data suggest that a mutation in the β-myosin heavy chain causes noncompaction of the ventricular myocardium associated with a variable expressivity of the disease. The combination of an ‘old’ gene with a ‘new’ phenotype may help to unravel causes, mechanisms and clinical implications of NVM.

### Note added in proof

In the course of the review process, three different manuscripts have been published that link defects in sarcomeric proteins to noncompaction of the ventricular myocardium (Monserrat et al., European Heart Journal, 2007; Hoedemaekers et al., European Heart Journal, 2007; Kaneda et al., Clinical Science, 2007). However, the present work is the only one that shows linkage of the disease gene region to noncompaction in a genome-wide analysis.

## Materials and Methods

### Clinical evaluation

The clinical and genetic studies were carried out according to institutional guidelines after approval of the local ethics committee (Ethics Committee of the University of Witten/Herdecke) and given informed consent from all participants. Twenty four members of family DU-11 were clinically evaluated by a review of their medical history, physical examination, 12-lead echocardiography (ECG) and 2-dimensional transthoracic echocardiography (TTE). Echocardiographic measurements of wall thickness and cavity dimensions were performed using M-mode and 2-dimensional views. NVM was identified based on the following diagnostic criteria [12], [25]: Presence of prominent multiple trabeculations, deep recesses in the thickened myocardium filled with blood from the ventricular cavity (as visualized by color Doppler imaging), presence of a two layered structure of the endomyocardium with a thin compacted layer and a thick non-compacted layer. The NVM affection status was classified as follows: unknown, deceased individuals and/or no clinical records available; unaffected, absence of a non-compacted myocardial layer; partially affected, documented apical thickening (<18 mm) due to noncompaction with a ratio of non-compacted/compacted myocardium (N/C ratio) of either <2 or not quantifiable; affected, apical thickening (>18 mm) due to noncompaction with a N/C ratio ≥2. We note, that neither the partially affected nor the affected patients showed signs of canonical hypertrophic cardiomyopathy (see Results section). The NVM status of individual III:8 was confirmed by magnetic resonance tomography (MRT). Ebstein's anomaly was diagnosed echocardiographically based on the dislocation of the septal leaflet of the tricuspid valve from the insertion of the anterior leaflet of the mitral valve towards the apex by ≥8 mm/m^2^ body surface area [26]. EDTA-blood was taken from 24 members of family DU-11, and DNA was available from 184 healthy volunteers.

### Linkage analysis and sequencing

DNA was extracted from peripheral blood samples using standard methods. A genome-wide scan with a set of 430 polymorphic microsatellite markers (modified Weber9-set, Marshfield Institute), average spacing of about 10 cM, was performed. Additional markers including two microsatellites in the *MYH7* gene (one in the promotor region, *MYO I*, and one in intron 24, *MYOII* (ref. [27], [28]) , were used for fine mapping. Products of PCR assays with fluorescently labeled primers were analyzed by automated capillary genotyping on a MegaBACE 1000 (GE Healthcare Biosciences, Piscataway, NJ) and scored using the Genetic Profiler analysis software (GE Healthcare Biosciences, Piscataway, NJ). A two-point linkage analysis was performed using the program SuperLink of easyLINKAGE Plus (version 5.02) [29] assuming an autosomal dominant model of disease inheritance with a disease allele frequency of 0.001, equal female and male recombination rates and two liability classes. The penetrance values were set at 0.9, 0.8 or 0.7 for the affecteds (liability class 1) and at 0.5 for the partially affected (II:3 and IV:6), respectively. Haplotypes were reconstructed using SIMWALK2 (version 2.91) [30]. Bidirectional sequencing of all coding exons and splice sites of the human genes *MYH6* and *MYH7* was carried out for the index patient III:4 of family DU-11. The primer sequences were obtained on the basis of reported nucleotide sequences [31] and the Genbank entry NT_026437. Sequencing was performed according to the Dye Terminator Cycle Sequencing Quick Start Kit protocol (Beckman Coulter, Fullerton, CA) on a CEQ 8000 XL DNA Sequencing System (Beckman Coulter, Fullerton, CA). Sequences were analyzed and aligned using the CEQuence Investigator program (Beckman Coulter, Fullerton, CA). In the other members of the family only *MYH7* exon 10 was sequenced to determine the occurrence of the R281T mutation. Healthy controls were analyzed by pyrosequencing for the presence or absence of the R281T mutation in *MYH7* exon 10. Pyrosequencing was performed as described previously [32].

### Protein modeling

Protein models were derived from the RCSB Protein Data Bank (see below). The ribbon plots were drawn using the program ViewerPro version 4.2 (Accelrys Inc., San Diego, CA) and PyMOL Molecular Graphics System (Delano Scientific, San Carlos, CA).

### Protein alignment and accession numbers

Multiple sequence alignments were performed with the ClustalW program.

Genbank: *Homo sapiens* chromosome 14 genomic contig, reference assembly (*MYH6* and *MYH7*), NT_026437; *Homo sapiens* heavy polypeptide 7, cardiac muscle, beta (MYH7), NM_000257; RCSB Protein Data Bank (PDB): chicken skeletal muscle myosin subfragment-1, 2MYS; UniProtKB: human myosin heavy chain, cardiac muscle beta isoform, P12883; SwissProt entry names: MYH7_HUMAN-human myosin heavy chain, cardiac muscle beta isoform; MYH1_HUMAN-human myosin heavy chain, skeletal muscle, adult 1; MYH4_HUMAN-human myosin heavy chain, skeletal muscle, fetal; MYH1_MOUSE-murine myosin heavy chain, skeletal muscle, adult 1; MYH4_RABIT-rabbit myosin heavy chain, skeletal muscle, juvenile; MYSS_CHICK-chicken myosin heavy chain, skeletal muscle, adult; Q802Z4_BRARE (Q802_BRARE)-zebrafish protein Q802Z4; MYSA_DROME-fruit fly myosin heavy chain, muscle; MYO3_CAEEL-*Caenorhabditis elegans* myosin heavy chain A.

### Web Resources

URLs for data presented herein are as follows:

GenBank, http://www.ncbi.nih.gov/Genbank/index.html

RCSB Protein Data Bank, http://www.rcsb.org/pdb/Welcome.do

UniProtKB/Swiss-Prot DATABASE, http://www.ebi.ac.uk/swissprot/

OMIM, http://www.ncbi.nlm.nih.gov/Omim/
